# Exploring the Sociodemographic and Health-Related Determinants of Telehealth Use Among a Cohort of Older Australians During the COVID-19 Pandemic: Repeated Cross-Sectional Study

**DOI:** 10.2196/58594

**Published:** 2024-08-23

**Authors:** Shanna Fealy, Suzanne McLaren, Claire Ellen Seaman, Melissa Nott, Donovan Jones, Pauletta Irwin, Patricia Logan, Rachel Rossiter, Simon McDonald

**Affiliations:** 1 School of Nursing, Paramedicine and Healthcare Sciences Faculty of Science and Health Charles Sturt University Port Macquarie Australia; 2 School of Medicine and Public Health College of Health, Medicine and Wellbeing The University of Newcastle Australia Callaghan Australia; 3 Ageing Well in Rural and Regional Australia Research Group Charles Sturt University Albury Australia; 4 Ageing Well Research Group Charles Sturt University Albury Australia; 5 School of Psychology Faculty of Business, Justice and Behavioural Sciences Charles Sturt University Port Macquarie Australia; 6 Three Rivers Department of Rural Health Charles Sturt University Albury Australia; 7 School of Dentistry and Medical Sciences Faculty of Science and Health Charles Sturt University Bathurst Australia; 8 School of Rural Medicine Faculty of Science and Health Charles Sturt University Orange Australia; 9 The Spatial Data Analysis Network Charles Sturt University Port Macquarie Australia

**Keywords:** telehealth, telemedicine, aging, older people, COVID-19, Australia, 45 and Up Study, health-related determinates, Technology Acceptance Model, mobile phone

## Abstract

**Background:**

During the COVID-19 pandemic, there was a rapid adoption of telehealth care services as a public health strategy to maintain access to essential health care. In Australia, there has been increasing optimism for the expansion of telehealth services. However, little is known about the patterns and determinants of telehealth adoption among older adults, with concerns that an expansion of telehealth services may only be of benefit to those who already have better access to health care.

**Objective:**

Leveraging data collected by The Sax Institute’s 45 and Up COVID Insights study between November 2020 and April 2022, the objective of this study was to identify and describe the sociodemographic and health-related determinants of telehealth adoption and use among a cohort of older Australians. We hypothesized that health-related factors would be key determinants of telehealth adoption for Australians aged ≥65 years during the COVID-19 pandemic.

**Methods:**

A repeated cross-sectional design was used. The relationships between telehealth use (classified as low, moderate, or high) and selected sociodemographic and health-related characteristics were assessed using logistic regression techniques. Variable selection and findings were situated within the Technology Acceptance Model, the Unified Theory of Acceptance, and the Use of Technology theoretical frameworks.

**Results:**

Of the 21,830 participants aged ≥65 years, the proportion who indicated adopting telehealth ranged from 50.77% (11,082/21,830) at survey 1 in 2020 to 39.4% (7401/18,782) at survey 5 in 2022. High levels of telehealth use were associated with being female, aged <85 years, living in a major city, cohabiting with others, and being from the most socioeconomically disadvantaged areas (deciles 1-3). Individuals with a disability, chronic disease, multimorbidity, and lower perceived quality of life and those experiencing missed or delayed care were significantly more likely to use telehealth across all levels (*P*<.001). A temporal association was observed, whereby participants who engaged with telehealth services before or early in the pandemic (as assessed in survey 1) were more likely to continue telehealth use when assessed in survey 5 in 2022 (*P*<.001).

**Conclusions:**

This research contributes to the broader understanding of telehealth adoption and use among older adults. As telehealth models of care expand, there is an opportunity to tailor these services to the needs of older adults, particularly those living with chronic diseases and multimorbidity, by using targeted strategies that overcome barriers to accessing specialized health care services.

## Introduction

### Overview

The COVID-19 pandemic triggered a rapid transformation in the delivery of health care. Because of adaptive necessity, in-person visits to health care providers were augmented through the widespread adoption of digital technology mediums [1]. In countries such as Australia, telehealth was incentivized as a national public health strategy to facilitate remote patient and practitioner interactions, given social distancing and lockdown measures [2-4]. Noting that telehealth and telemedicine are often used interchangeably within the literature [5], this paper uses telehealth to denote the delivery of health care services at a distance by health care professionals for remote patient engagement (synchronous or asynchronous), using information and communication technologies (ICTs), such as telephone or video mediums [6].

Before the COVID-19 pandemic, telehealth services in countries with high income, such as Australia, Canada, New Zealand, and the United States, had been established to facilitate access to health care services across vast geographic distances [2,7]. The sustained integration and adoption of telehealth services pose ongoing challenges, as evidenced by a notable decline in use after the acute pandemic phase [8,9]. Barriers to telehealth integration and adoption are well documented [10]. These include health care consumer and provider resistance and skepticism [8,10], concerns over patient safety (inability to undertake hands-on assessments) [8], confidentiality and privacy of medical information [4,6,10], a lack of digital technology infrastructure [8,10], socioeconomic disadvantage [4,6,11], varying levels of digital technology literacy [10], increasing age [4,6,8,10,12], education levels [10], and poorly designed platforms [10]. Since the start of the pandemic, there has been increasing optimism for the expansion of telehealth services to increase equity of health care access for those living outside of metropolitan areas in which poor health outcomes are increased. This is particularly relevant given the backdrop of the ever-increasing prevalence of noncommunicable chronic disease, multimorbidity, and aging populations [1,6,8,13,14]. A central concern is whether the expansion of telehealth services will genuinely improve health care access or disproportionately benefit those who already have better access to health care [4,6,15-17].

### Background

“The digital divide” has become increasingly prevalent in discussions around technology use, including telehealth [4,12]. The term encompasses the socioeconomic, ICT resource-related, and other accessibility disparities and their impacts on vulnerable groups. These groups typically include people of cultural and linguistic diversity (CALD); those with chronic disease, multimorbidity (defined as having more than one chronic disease), or disability; older adults; those living in rural and remote geographic areas; and those of lower income [4,12]. A recent systematic review by Haimi and Gesser-Edelsburg [6] highlighted a gap in the literature regarding telehealth engagement among older adults (aged ≥65 years) during the COVID-19 pandemic. Of the 11 reviewed studies, it was evident that although telehealth service availability increased, telehealth adoption and participation among older adults was low [6]. This is particularly concerning given that older people were more likely to develop severe disease, with those experiencing multimorbidity at even higher risk, emphasizing the need for further studies to identify challenges with telehealth adoption among this age group [6].

In Australia, where 1 in every 6 people is aged ≥65 years and where a significant proportion reside outside of metropolitan areas, challenges with the equitable provision of health care services are exacerbated [18-21]. There is considerable demand among policy makers for upscaled telehealth service models, particularly given the distinctive characteristics of older Australians [20]. A total of 50% of people aged ≥65 years are affected by disability [18], whereas 85% have at least 1 chronic disease, with 60% reporting multimorbidity [14]. For these people, overcoming barriers to telehealth adoption becomes imperative.

Dykgraaf et al [4] suggest that the adoption of telehealth by older Australians extends beyond conventional barriers, such as personal socioeconomics, ICT ownership, and internet or network connectivity issues. They propose that barriers to the adoption of telehealth involve a more nuanced interplay of factors related to digital literacy rather than digital deprivation, as well as being influenced by practical factors related to physical and cognitive health, trust and familiarity with technology, and ease of use, particularly during the COVID-19 pandemic [4]. Broader research suggests that for older people, attitudes, perceptions, and experience of digital technologies influence their intention to adopt these mediums, with characteristics such as age, gender, education, health status, social influences, and income identified as influencing (barriers and enablers) factors [15,22,23].

In this context, the Technology Acceptance Model provides a valuable framework and lens for examining the factors that determine an individual’s potential acceptance or rejection of technologies, including telehealth [24]. Originally developed by Davis [25], the Technology Acceptance Model is an adaption of the well-established Theory of Reasoned Action [24]. Although the Theory of Reasoned Action primarily focuses on understanding the motivational factors and determinates of health-related behaviors [26], the Technology Acceptance Model was tailored to elucidate the influencing factors related to information technology use [24,27]. Grounded in the concept that people tend to engage in behaviors that have a positive effect, the Technology Acceptance Model posits that perceived usefulness and perceived ease of use are the main determining factors underlying technology adoption. These factors are influenced by antecedent personal factors (attitudes) that impact behavioral intention and actual technology use [24]. The Unified Theory of Acceptance and Use of Technology, a recent extension of the Technology Acceptance Model, explains the individual influencing factors related to technology use across four constructs: (1) performance expectancy, the degree to which an individual believes the technology with help them; (2) effort expectancy, the degree of perceived ease of use; (3) social influence, the degree to which an individual perceives how others view the importance of using the technology is for them; and (4) facilitating conditions, the degree to which the individual believes that infrastructure exists to support their use of the technology [28].

The circumstances surrounding the COVID-19 pandemic present a unique opportunity to observe the real-world dynamics in telehealth adoption at the population level. The expansion of services and forced adoption of telehealth acts as a natural experiment for exploration, whereby external circumstances dictate the implementation of an intervention [29]. Mao et al [15] analyzed data from the Irish Longitudinal Study on Aging collected during the COVID-19 pandemic (COVID-19 wave, June to November 2020). Their findings suggest health-related factors (chronic conditions, multimorbidity, and poor mental health) and, to a lesser extent, sociodemographic factors (younger age [<70 years] and socioeconomic disadvantage) are determinants of telehealth use. A study conducted by Choi et al [12] analyzing data from the United States National Health and Aging Trend Study-COVID supplement (collected in 2020) similarly observed health-related factors (chronic conditions, impairments with activities of daily living, and moderate levels of mental distress) and younger age (<80 years) as determinants of telehealth use [10].

Leveraging data collected by The Sax Institute’s 45 and Up COVID Insights substudy, the objective of this study was to identify and describe the sociodemographic and health-related determinants of telehealth adoption and use among a cohort of older Australians. Consistent with the Technology Acceptance Model and Unified Theory of Acceptance and Use of Technology, we hypothesized that health-related factors will be key determinants of telehealth adoption and use for older Australians during the COVID-19 pandemic. These findings will contribute to our understanding of factors that influence telehealth adoption beyond conventional barriers for older Australians, working toward equity of health service provision for this population [4,28].

## Methods

### Study Design

This study uses survey data collected by The Sax Institute’s 45 and Up COVID Insights substudy (hereon referred to as COVID Insights study). Conducted in the state of New South Wales, Australia, between November 2020 and April 2022 [30], the COVID Insights study is an extension of the larger 45 and Up prospective longitudinal cohort study. The COVID Insights study covers critical themes addressing health care use, mental health and well-being, financial aspects, COVID-19 preventive measures, and lifestyle behaviors.

### Population

The Sax Institute’s 45 and Up Study, initiated between 2005 and 2009, has recruited a total of 267,357 participants from New South Wales [31]. Recruitment methods have been previously published [31]. Prospective participants were drawn at random from the Services Australia Medicare enrollment database, with those aged ≥80 years and residents of rural and remote areas intentionally oversampled [31]. About 19% of those invited to the study consented to participate. Consenting participants were followed up every 5 years in waves. Because of the large number of participants, surveys were also distributed in phases. For example, the wave-2 follow-up surveys were distributed in 4 phases between 2012 and 2015, with wave-3 follow-up surveys distributed across 3 phases between 2018 and 2020 [31].

The 45 and Up COVID Insights study was established in 2020 through collaborative efforts with key health industry stakeholders and policy makers and engaged 32,115 participants between November 2020 and April 2021 [30]. The cohort was formed through 2 distinct recruitment methods [30,32]. Method 1 involved reaching out to existing 45 and Up Study participants (n=85,299) who were completing their routine wave-3 (phase 3) follow-up survey. During the period between July and December 2020, these participants were asked to complete an additional COVID supplement survey, resulting in 28,840 participants expressing interest in the COVID Insights study [30,32]. From this group, 15,252 completed both the COVID supplement survey (which featured variables similar to the COVID Insights survey 1) and COVID Insights survey 2, officially joining the substudy at this time point [30,32].

**Figure 1:**
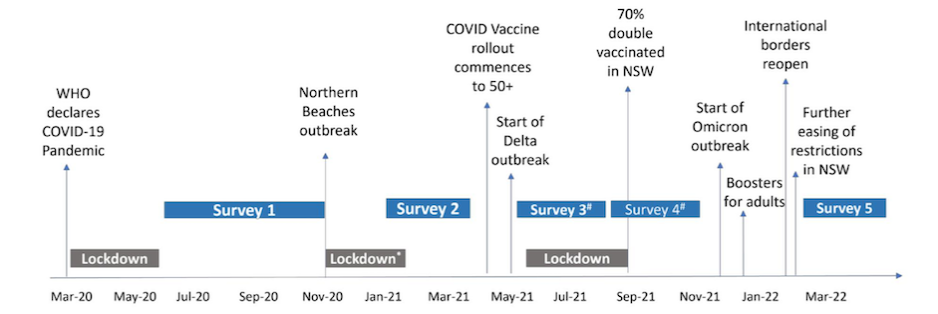
COVID Insights Survey administration, pandemic outbreaks, and pandemic policy implementation in New South Wales, Australia. The number sign (#) from surveys 3 and 4 indicates that data were collected in 3 consecutive month-long tranches. NSW: New South Wales; WHO: World Health Organization. *Lockdown in place across Sydney’s Northern Beaches with some restrictions for Greater Sydney.

Method 2 encompassed the distribution of invitations to a random sample of existing 45 and Up Study participants (n=60,000) between November and December 2020. Of these invitations, 40,000 were sent by email and 20,000 were distributed through traditional mail-in postal procedures. A total of 16,863 participants responded, completed the COVID Insights survey 1, and were successfully recruited to the substudy [30,32]. Five COVID Insights surveys were developed for rapid data collection and were strategically administered over various pandemic time points between 2020 and 2022, as displayed in Figure 1 [33]. All follow-up surveys (COVID Insights surveys 2-5) were exclusively administered via the web.

### Participants

Of the 32,115 participants available for analysis, 21,830 participants aged ≥65 years who completed telehealth adoption questions were identified. This subset was chosen for the current analysis as it aligns with the Australian Institute of Health and Welfare’s age criterion for older adults [21] and is supported by the age classification used in the systematic review by Haimi and Gesser-Edelsburg [6] for ease of comparison across data sets.

### Variable Selection

Using a repeated cross-sectional design and guided by previous research and the Technology Acceptance Model and Unified Theory of Acceptance and Use of Technology frameworks, a comprehensive set of sociodemographic and health-related variables was considered for analysis. Specifically, these frameworks helped us to select a set of variables aimed at understanding the individual influencing factors related to perceived usefulness, ease of use, performance expectancy, effort expectancy, social influence, and facilitating conditions [28]. Therefore, we included the following variables: age, gender, CALD, disability status, chronic disease and multimorbidity status, carer status, housing type, number of people in the household, geographic location (as per the Accessibility/Remoteness Index of Australia [34]), socioeconomic disadvantage (as per the Socioeconomic Indexes For Areas [35]), information related to cigarette smoking and alcohol consumption, vehicle driving, experiences of missed or delayed health care, Kessler–6 psychological distress scores [36], De Jong Gierveld Loneliness scores [37], and general quality of life ratings.

### Sociodemographic and Health-Related Variables

With the exception of smoking, alcohol consumption, vehicle driving, missed or delayed care, Kessler–6, De Jong Gierveld scores, and quality of life ratings, all other listed sociodemographic and health-related variables were packaged as static variables for the COVID Insights study [32]. This means they were either derived from the wider 45 and Up Study or collected as part of the COVID Insights survey series and do not change over time [32]. Participants were identified as people of CALD if they were born outside of Australia, New Zealand, the United Kingdom, Ireland, Canada, the United States, or South Africa. In addition, individuals who indicated speaking a language other than English at home were classified as people of CALD [32]. Chronic conditions were assessed by asking participants whether a doctor had ever told them that they had cancer, cardiovascular disease, stroke, diabetes, asthma, arthritis, Parkinson disease, or chronic kidney disease. With count data only supplied, multimorbidity was determined by the number of chronic diseases reported, indicating the presence of >1 concurrent chronic disease. Individuals residing in outer regional, rural, and very remote geographic localities were combined into one category because of the small samples of participants within these subgroups. The socioeconomic disadvantage area deciles, in which decile 1 equates to the most disadvantaged areas, were collapsed into 3 categories for ease of interpretation.

The missed or delayed care variable was derived from participant responses to whether they had experienced missed or delayed health care from a general practitioner visit, hospital visit, specialist doctor, or prescription medication visit. The mental health of participants was assessed using mean Kessler–6 scores. Kessler–6 scores range from 0 to 24; higher scale scores indicate more serious mental distress [36,38], with a cutoff point of ≥13 suggesting probable mental illness [38]. Mean De Jong Gierveld Loneliness (short form) scores were used. The scale comprised 3 positively framed questions and 3 negatively framed questions, with responses provided as “Yes,” “More or less,” or “No” [37]. Only positive (Yes) and neutral responses (More or less) to the 3 negatively framed questions are counted, resulting in scores ranging from 0 to 3, whereby 0 indicates no emotional loneliness and 3 suggests intense emotional loneliness [37]. Mean general quality of life scores were derived from responses to a 5-point rating scale, whereby lower scores indicate better quality of life (eg, 1=excellent and 5=poor).

### Telehealth Variables

Telehealth use was defined as the use of health care services via telephone or video call. Questions related to telehealth adoption and use were assessed in surveys 1, 4, and 5. In the initial survey (administered between July and December 2020), participants were asked whether they had used telehealth services since January 2020, which is defined as an appointment with a health care provider by video or telephone instead of an in-person visit. Participants were asked to specify whether the telehealth mode was via telephone or video call at this time point only. In survey 4 (administered between September and November 2021), participants were asked whether they had used telehealth (via telephone or video call) in the last 3 months and for which purposes (eg, a regular check-up, medical diagnosis or advice, initial consultation, follow-up consultation, receive test results, or treatment or therapy review). In survey 5 (administered between March and April 2022), participants were again asked whether they had used telehealth (via telephone or video call) in the past 3 months and which health care provider they saw (general practitioner, specialist doctor, pharmacist, nurse, mental health care [eg, psychologist or counselor], physiotherapist, occupational therapist, dietitian).

For this analysis, participants who indicated telehealth use across the 3 surveys were categorized on the basis of their individual level of telehealth use as follows: no adoption (did not use telehealth at any survey); low use (used telehealth at 1 of the 3 surveys only); moderate use (used telehealth at 2 surveys); and high use (used telehealth at all 3 surveys).

### Statistical Analysis

All sociodemographic, health-related, and telehealth-related variables were summarized and reported using descriptive statistics. To investigate the determinants of telehealth use (dependent variable), a multinomial logistic regression model was developed using the selected sociodemographic and health-related characteristics as independent variables. Data were pooled across all 3 surveys (surveys 1, 4, and 5). Participants with missing data on any of the analysis variables were excluded. An additional logistic regression analysis was conducted to explore the determinants of telehealth adoption, specifically investigating whether prior telehealth adoption, assessed in survey 1, predicted telehealth use in survey 5. All variables were checked for independence and multicollinearity, satisfying these assumptions with telehealth variables organized into mutually exclusive and exhaustive categories. All analyses were conducted by a statistician (SM) using R programming language (version 4.3.0; R Development Core Team) within The Sax Institute’s Secure Unified Research Environment. The criterion for statistical significance was set at the .05 α level.

### Ethical Considerations

For The 45 and Up Study, all participants provided written informed consent with approval obtained from the University of New South Wales, Human Research Ethics Committee (HC210602) [31]. The COVID Insights study was approved by the University of New South Wales, Human Research Ethics Committee (reference HC200597) [30] with approval for the current analysis also obtained by the authors institutional Human Research Ethics Committee (reference H23582).

## Results

### Sociodemographic, Health-Related, and Telehealth-Related Characteristics

Table 1 displays the sociodemographic and health-related characteristics of the cohort, categorized by telehealth use across surveys. Inspection of baseline sociodemographic and health-related characteristics at survey 1 (n=21,830) revealed most participants were women, were not people of CALD, lived in a house, lived with ≥1 other person, resided in a major city, and did not have carer responsibilities. Regarding health, most did not live with a disability but were impacted by chronic disease. Most had not experienced missed or delayed health care because of the COVID-19 pandemic. Mean Kessler–6 scores were <13, and mean De Jong Gierveld Loneliness scores were <2. The cohort reported relatively high levels of perceived quality of life. The cohort was assumed to have adequate levels of technology literacy as surveys 2 to 5 were administered electronically. When assessed at survey 4, most participants reported regular use of a smartphone (7166/7890, 90.82%) and computer device (6809/7890, 86.3%).

**Table 1:** Sociodemographic, health-related, and telehealth-related cohort characteristics^a^.

Variables				Survey 1 (n=21,830)			Survey 4 (n=18,268)				Survey 5 (n=18,782)		
				Used telehealth		Did not use telehealth	Used telehealth		Did not use telehealth		Used telehealth		Did not use telehealth
Cohort, n (%)				11,082 (50.77)		10,748 (49.23)	7890 (43.19)		10,378 (56.81)		7401 (39.4)		11,381 (60.6)
**Sociodemographic characteristics**													
	**Age group (y), n (%)**												
		65-74	7674 (69.25)		7516 (69.93)		5271 (66.81)	6891 (66.4)		4735 (63.98)		7451 (65.47)	
		75-84	3040 (27.43)		2878 (26.78)		2330 (29.53)	3104 (29.91)		2395 (32.36)		3481 (30.59)	
		>85	368 (3.32)		354 (3.29)		289 (3.66)	383 (3.69)		271 (3.66)		449 (3.95)	
	**Gender, n (%)**												
		Man	4777 (43.11)		5511 (51.27)		3423 (43.38)	5037 (48.54)		3266 (44.13)		5357 (47.07)	
		Woman	6305 (56.89)		5237 (48.73)		4467 (56.62)	5341 (51.46)		4135 (55.87)		6024 (52.93)	
	**Which of the following devices do you use regularly?, n (%)^b^**												
		A smartphone (Android, Apple, or other)	—^c^		—		7166 (90.82)	9205 (88.7)		—		—	
		A computer (Windows or Apple)	—		—		6809 (86.3)	8853 (85.31)		—		—	
		A tablet device (Apple or other tablets)	—		—		4607 (58.39)	5682 (54.75)		—		—	
		A wearable device (Fitbit, Garmin, Apple Watch, or other)	—		—		1769 (22.42)	2034 (19.6)		—		—	
	Cultural and linguistic diverse, n (%)			833 (7.52)		984 (9.16)	622 (7.88)		830 (8)		585 (7.9)		913 (8.02)
	Has carer responsibilities^d^, n (%)			1405 (12.68)		997 (9.28)	1005 (12.74)		1010 (9.73)		989 (13.36)		1086 (9.54)
	Drives a car, n (%)			—		—	—		—		5970 (80.66)		9277 (81.51)
	**Housing^e^, n (%)**												
		House or house on a farm	8817 (79.56)		8765 (81.55)		6198 (78.56)	8473 (81.64)		5843 (78.95)		9288 (81.61)	
		Flat, unit, apartment, or granny flat	1644 (14.83)		1529 (14.23)		1272 (16.12)	1434 (13.82)		1175 (15.88)		1585 (13.93)	
		Retirement village, self-care unit, hostel for the aged, or nursing home	515 (4.65)		362 (3.37)		353 (4.47)	361 (3.48)		319 (4.31)		399 (3.51)	
		Mobile home, temporary housing, or boarding house	58 (0.52)		48 (0.45)		34 (0.43)	65 (0.63)		39 (0.53)		64 (0.56)	
		Other	47 (0.42)		44 (0.41)		33 (0.42)	44 (0.42)		25 (0.34)		45 (0.4)	
	**Living arrangements^f^, n (%)**												
		Lives alone	2398 (21.64)		2291 (21.32)		1720 (21.8)	2303 (22.19)		1543 (20.85)		2462 (21.63)	
		Lives with 1 or more other persons	8577 (77.4)		8382 (77.99)		6170 (78.2)	8075 (77.81)		5843 (78.95)		8896 (78.17)	
	**ARIA^g,h^, n (%)**												
		Major city	6248 (56.38)		6002 (55.84)		5016 (63.57)	5437 (52.39)		4712 (63.67)		6085 (53.47)	
		Inner regional areas	4006 (36.15)		3788 (35.24)		2398 (30.39)	3987 (38.42)		2212 (29.89)		4312 (37.89)	
		Outer regional, remote, very remote areas	817 (7.37)		936 (8.71)		467 (5.92)	935 (9.01)		462 (6.24)		968 (8.51)	
	**Socioeconomic disadvantage^i^, n (%)**												
		1-3	2451 (22.12)		2041 (18.99)		1505 (19.07)	2133 (20.55)		1434 (19.38)		2312 (20.31)	
		4-7	4026 (36.33)		4004 (37.25)		2821 (35.75)	3894 (37.52)		2656 (35.89)		4207 (36.97)	
		8-10	4476 (40.39)		4588 (42.69)		3474 (44.03)	4235 (40.81)		3220 (43.51)		4733 (41.59)	
**Health-related characteristics**													
	**Alcohol intake^j^, n (%)**												
		Consumers	4674 (42.18)		4447 (41.38)		—	—		5607 (75.76)		8932 (78.48)	
		Nonconsumers	1449 (13.08)		1226 (11.41)		—	—		1794 (24.24)		2449 (21.52)	
	**Smoking Status n (%)**												
		Smokers	—		—		—	—		99 (1.34)		179 (1.57)	
		Nonsmokers	—		—		—	—		7302 (98.66)		11,202 (98.43)	
	Has a disability^k^, n (%)			638 (5.76)		231 (2.15)	389 (4.93)		229 (2.21)		367 (4.96)		244 (2.14)
	Has a chronic condition, n (%)			3590 (32.39)		4068 (37.85)	2563 (32.48)		3929 (37.85)		2360 (31.89)		4321 (37.97)
	Has multimorbidity^l^, n (%)			6186 (55.83)		4636 (4313)	4398 (55.74)		4538 (43.73)		4205 (56.82)		4954 (43.53)
	**Missed or delayed health care^m^, n (%)**												
		Experienced missed or delayed care	1579 (14.25)		970 (9.02)		1079 (13.68)	951 (9.16)		841 (11.36)		768 (6.75)	
		Did not experience missed or delayed care	4544 (41.00)		4703 (43.76)		1109 (14.06)	1270 (12.24)		538 (7.27)		651 (5.72)	
	Kessler–6 (psychological distress) scale^n,o^, mean (SD)			8.32 (3.03)		7.75 (2.59)	—		—		9.32 (3.46)		8.63 (3.13)
	De Jong Gierveld Loneliness Scale^p,q^, mean (SD)			1.96 (1.66)		1.82 (1.6)	—		—		2.02 (1.77)		1.77 (1.67)
	Rate quality of life^r,s^, mean (SD)			2.09 (0.79)		1.94 (0.74)	2.23 (0.87)		2.04 (0.81)		2.22 (0.83)		2.04 (0.76)

^a^All values in the table have been rounded to two decimal places nearest as such percentages may not sum up to exactly 100% because of rounding error and missing data.

^b^Missing: survey 4 (n=272).

^c^Responses to these questions were not elicited at these timepoints and reflect the change in survey questions overtime.

^d^Missing: survey 1 (n=1) and survey 5 (n=1).

^e^Missing: survey 1 (n=1) and survey 4 (n=1).

^f^Missing: survey 1 (n=182) and survey 5 (n=38).

^g^ARIA: Accessibility Remoteness Index of Australia.

^h^Missing: survey 1 (n=33), survey 4 (n=28), and survey 5 (n=31).

^i^Socioeconomic disadvantage lower rankings indicate most disadvantaged.

^j^Missing: survey 1 (n=10,034).

^k^Missing: survey 1 (n=14), survey 4 (n=9), and survey 5 (n=7).

^l^Multimorbidity refers to those who have indicated having >1 chronic disease.

^m^The missed or delayed care variable includes those indicating missed or delayed care pertaining to a visit to a general practitioner, hospital, specialist, and prescription medication (missing survey 1, n=10,034; survey 4, n=13,859; and survey 5, n=15,984).

^n^Kessler–6—scores are summarized on a scale of 0-24, with higher scores indicating greater distress; scores ≥13 indicating probable mental illness.

^o^Missing: survey 1 (n=266).

^p^De Jong Gierveld Loneliness Scale is scored on a scale of 0-3, with scores of 3 indicating intense emotional loneliness and 0 indicating no loneliness.

^q^Missing: survey 1 (n=230).

^r^Quality of life rating scores are based on a Likert scale of 1-5 (1=excellent; 2=very good; 3=good; 4=fair; 5=poor).

^s^Missing: survey 1 (n=10,034).

A decreasing trend in telehealth adoption was evident across survey time points. Adoption was highest at survey 1 in 2020, with 51.77% (11,082/21,830) of the cohort using telehealth services (telephone or video), indicative of both prepandemic and early pandemic adoption (official announcement of the pandemic in March 2020, with lockdown measures occurring up until May 2020). The proportion of users had decreased at survey 4 to 43.19% (7890/18,268), capturing use in response to the COVID-19 Delta outbreak and associated lockdowns occurring in 2021. The lowest levels of use were observed in survey 5, with only 39.4% (7401/18,782) of participants using telehealth at this time point (capturing use following the vaccination rollout in 2022). As per Table 2, when assessed in survey 4, a total of 42.72% (3371/7890) of participants indicated that they were more likely to use telehealth for a follow-up consultation or to receive test results (2901/7890, 36.81%). A smaller proportion (1608/7890, 20.38%) indicated using telehealth for initial health care consultations or to receive a medical diagnosis or advice (1693/7890, 21.46%). By survey 5, telehealth was primarily being used for general practitioner appointments (5634/7401, 76.12%) or specialist health care appointments (1421/7401, 19.2%), with most participants (4629/7401, 62.55%) preferring a hybrid health care model involving both telehealth and in-person care.

**Table 2:** Telehealth-related cohort characteristics^a^.

Telehealth-related characteristics		Used telehealth		
		Survey 1 (n=11,082)	Survey 4 (n=7890)	Survey 5 (n=7401)
**Mode of telehealth service, n (%)^b^**				
	Telephone	9857 (88.95)	—^c^	—
	Video	350 (3.16)	—	—
	Both	706 (6.37)	—	—
**How likely would you be to recommend telehealth services to someone else?, n (%)^d^**				
	Definitely will not	371 (3.35)	—	—
	Probably will not	2676 (24.15)	—	—
	Probably will	5587 (50.42)	—	—
	Definitely will	2320 (20.93)	—	—
**How useful do you think it will be to have appointments via telehealth after the COVID-19 emergency is over?, n (%)^e^**				
	Not at all	1257 (11.34)	—	—
	Slightly	2205 (19.9)	—	—
	Moderately	3869 (34.91)	—	—
	Very	2759 (24.9)	—	—
	Extremely	976 (8.81)	—	—
**In the last 3 months have you used telehealth for any of the following? (Participants could choose multiple options), n (%)**				
	Regular check-up	—	1158 (14.68)	—
	Medical diagnosis or advice	—	1693 (21.46)	—
	Initial consultation	—	1608 (20.38)	—
	Follow-up consultation	—	3371 (42.72)	—
	Receive test results	—	2904 (36.81)	—
	Treatment or therapy	—	447 (5.67)	—
	Treatment or therapy review	—	848 (10.75)	—
	Other	—	788 (9.99)	—
**Thinking about your latest telehealth appointment, which health provider did you see on this occasion?, n (%)**				
	General practitioner	—	—	5634 (76.12)
	Specialist doctor	—	—	1421 (19.2)
	Pharmacist	—	—	16 (0.22)
	Nurse	—	—	57 (0.77)
	Mental health care	—	—	143 (1.93)
	Physiotherapist or occupational therapist	—	—	46 (0.62)
	Dietician	—	—	17 (0.23)
	Other	—	—	67 (0.91)
**What is your preferred way of receiving this type of care?, n (%)**				
	I would prefer to have all of this type of care via telehealth	—	—	178 (2.41)
	I would prefer to have some of this type of care via telehealth and some face-to face	—	—	4629 (62.55)
	I would prefer to have all of this type of care face-to-face	—	—	2263 (30.58)
	I do not have a preference	—	—	331 (4.47)
**How did your latest telehealth service compare to a traditional in-person medical visit?, n (%)**				
	Better than a traditional visit	—	—	207 (2.8)
	Just as good as a traditional visit	—	—	4765 (64.38)
	Worse than a traditional visit	—	—	1893 (25.58)
	Not sure	—	—	536 (7.24)

^a^All values in the table have been rounded to the nearest whole percent, as such percentages may not sum up to exactly 100% because of rounding errors, missing data, and options in which multiple responses could be provided.

^b^Missing: survey 1 (n=169).

^c^Responses to these questions were not elicited at these timepoints and reflect the change in survey questions overtime.

^d^Missing: survey 1 (n=128).

^e^Missing: survey 1 (n=16).

### Logistic Regression Models

The results of the multinomial regression model, identifying sociodemographic and health-related determinates of telehealth use, are presented in Table 3. Women were more likely to engage with telehealth services across all categories (low, moderate, and high) compared with men (*P*<.001), with odds ratios indicating an increasing likelihood of use. Individuals aged ≥85 years were significantly (*P=*.01) less likely to exhibit high telehealth use compared with the reference group (aged 65-74 years)*.* Living alone was associated with lower odds of moderate (*P*=.002) and high (*P*<.001) telehealth use when compared with living with others. Inner regional residents and those in outer regional, rural, and very remote areas were less likely to use telehealth at moderate and high levels (*P*<.001) compared with major city dwellers. Individuals from areas of socioeconomic disadvantage (deciles 1-3) were more likely to use telehealth than those with lower levels of socioeconomic disadvantage (deciles 8-10; *P*=.02).

**Table 3:** Multinomial logistic regression model of sociodemographic and health-related determines of telehealth use (n=10,518)^a^.

Demographic and psychosocial characteristics				Coefficient		*Z* score		*P* value		Odds ratio (95% CI)
**Female (reference: male)**										
	Low^b^			0.26		4.69		<.001		1.30 (1.16-1.45)
	Moderate			0.39		6.69		<.001		1.48 (1.32-1.66)
	High			0.44		7.13		<.001		1.55 (1.38-1.75)
**Age (y; reference: 65-74 y)**										
	**75-84**									
		Low^b^	0.04		0.68		.50		1.04 (0.92-1.17)	
		Moderate	–0.09		–1.41		.16		0.91 (0.80-1.04)	
		High	–0.09		–1.31		.19		0.92 (0.43-0.87)	
	≥**85 y**									
		Low^b^	–0.12		–0.80		.43		0.89 (0.66-1.19)	
		Moderate	–0.24		–1.46		.14		0.79 (0.58-1.08)	
		High	–0.49		–2.77		.01		0.61 (0.43-0.87)	
**Smokers (reference: nonsmokers)**										
	Low^b^			–0.34		–1.54		.12		0.71 (0.46-1.10)
	Moderate			–0.57		–2.32		.02		0.57 (0.35-0.92)
	High			–0.47		–1.93		.05		0.63 (0.39-1.01)
**Alcohol consumers (reference: nonconsumers)**										
	Low^b^			0.07		–1.03		.30		0.94 (0.83-1.06)
	Moderate			–0.01		–0.10		.92		0.99 (0.87-1.13)
	High			–0.10		–1.41		.16		0.91 (0.79-1.04)
**Has a disability (reference: no disability)**										
	Low^b^			0.43		2.10		0.04		1.53 (1.03-2.29)
	Moderate			0.70		3.51		<.001		2.01 (1.36-2.96)
	High			1.12		5.87		<.001		3.06 (2.11-4.45)
**Has a chronic disease (reference: no chronic disease)**										
	Low^b^			0.16		2.15		.03		1.17 (1.01-1.36)
	Moderate			0.31		3.72		<.001		1.36 (1.16-1.60)
	High			0.54		5.65		<.001		1.72 (1.43-2.08)
**Has multimorbidity (reference: no multimorbidity)**										
	Low^b^			0.45		5.98		<.001		1.57 (1.35-1.82)
	Moderate			0.84		10.17		<.001		2.32 (1.97-2.73)
	High			1.29		13.63		<.001		3.63 (3.01-4.36)
**Lives alone (reference: lives ≥1 other person)**										
	Low^b^			–0.12		–1.88		.06		0.88 (0.78-1.01)
	Moderate			–0.22		–3.08		.002		0.81 (0.70-0.92)
	High			–0.33		–4.41		<.001		0.72 (0.62-0.83)
**Vehicle drivers (reference: nondrivers)**										
	Low^b^			–0.01		–0.12		.90		0.99 (0.87-1.14)
	Moderate			–0.06		–0.85		.39		0.94 (0.82-1.08)
	High			0.12		1.58		.11		1.13 (0.97-1.31)
**Remoteness (reference: major city)**										
	**Inner regional**									
		Low^b^	–0.03		–0.58		.56		0.97 (0.86-1.09)	
		Moderate	–0.37		–5.91		<.001		0.69 (0.61-0.78)	
		High	–0.61		–9.04		<.001		0.54 (0.48-0.62)	
	**Outer regional, rural, or very remote**									
		Low^b^	–0.06		–0.61		.54		0.94 (0.77-1.15)	
		Moderate	–0.51		–4.49		<.001		0.60 (0.48-0.75)	
		High	–0.93		–7.29		<.001		0.40 (0.31-0.51)	
**Relative socioeconomic disadvantage (SEIFA^c^ deciles) (reference deciles 1-3)**										
	**4-7**									
		Low^b^	–0.10		–1.35		.18		0.91 (0.78-1.05)	
		Moderate	–0.12		–1.57		.12		0.88 (0.76-1.03)	
		High	–0.09		–1.14		.25		0.91 (0.77-1.07)	
	**8-10**									
		Low^b^	–0.11		–1.41		.16		0.90 (0.77-1.04)	
		Moderate	–0.14		–1.69		.09		0.87 (0.74-1.02)	
		High	–0.19		–2.27		.02		0.82 (0.70-0.97)	
**Experienced missed or delayed health care (reference no missed delayed care)**										
	Low^b^			0.22		3.87		<.001		1.25 (1.12-1.40)
	Moderate			0.32		5.38		<.001		1.38 (1.23-1.56)
	High			0.53		8.51		<.001		1.70 (1.50-1.92)
**Kessler 6 (psychological distress)^d^**										
	Low^b^			0.02		1.86		.06		1.02 (1.00-1.05)
	Moderate			0.03		2.47		.01		1.03 (1.01-1.06)
	High			0.07		4.96		<.001		1.07 (1.04-1.10)
**De Jong (loneliness) scores^d^**										
	Low^b^			–0.06		–2.46		.01		0.95 (0.91-0.99)
	Moderate			–0.05		–2.30		.02		0.95 (0.90-0.99)
	High			–0.03		–1.19		.23		0.97 (0.93-1.02)
**Quality of life scores^d^**										
	Low^b^			0.14		2.87		.004		1.15 (1.05-1.26)
	Moderate			0.25		4.95		<.001		1.28 (1.16-1.42)
	High			0.31		5.88		<.001		1.36 (1.23-1.51)

^a^*Z* scores (Wald *z* test).

^b^Reference category for telehealth use was 0=did not use telehealth at all.

^c^SEIFA: Socioeconomic Indexes For Areas.

^d^Mean scores were derived from repeated measures.

In terms of health-related determinates, the presence of a disability, chronic disease, or multimorbidity was positively associated with all levels of telehealth use, with a clear trend of increasing odds from low to high levels observed. Experiences of missed or delayed health care were linked to higher odds of using telehealth across all levels. Higher levels of psychological distress were related to an increased likelihood of moderate (*P*=.01) and high (*P*<.001) telehealth use. An inverse relationship between loneliness and moderate (*P*=.02) and low levels (*P*=.01) of telehealth use was evident, with lower loneliness scores associated with higher odds of telehealth use. A lower quality of life was significantly associated with telehealth adoption across all levels (*P*=.004 to *P*<.01), with a notable increase in odds from low to high levels observed.

As displayed in Table 4, a strong positive association was found between early telehealth adoption (measured in survey 1) and subsequent telehealth use (measured in survey 5; *P*<.001). Having a chronic condition (*P*=.006) or multimorbidity (*P*<.001) was significantly associated with ongoing telehealth use at survey 5. The interaction between early telehealth adoption and having a chronic condition or multimorbidity was not significant.

**Table 4:** Survey effect, telehealth adoption, and ongoing use.

Telehealth use in survey 5 is predicted by?			Coefficient		*Z* score		*P* value
Telehealth adoption at S1^a^			1.11		12.15		<.001
Has a chronic condition			0.21		2.76		.006
Has multimorbidity			0.57		7.81		<.001
Has a disability			0.63		0.72		.47
**Interaction effects**							
	Telehealth adoption at S1^a^ and chronic conditions	0.05		0.51		.61	
	Telehealth adoption at S1 and multimorbidity	0.01		0.08		.93	

^a^S1: survey 1.

## Discussion

### Principal Findings

We aimed to investigate the sociodemographic and health-related determinants influencing telehealth adoption and use among a cohort of older Australians during the COVID-19 pandemic. The objective was to contribute to a comprehensive understanding of the factors influencing adoption and use, surpassing traditional barriers related to ICT ownership and access and motivated by a central concern that the expansion of telehealth services outside of pandemic conditions may exacerbate health care disparities.

Our analyses have revealed a unique set of determinants related to varying levels of telehealth use, suggesting a more nuanced interplay of factors that extend beyond sociodemographic determinants [4]. High telehealth use was significantly more prevalent among women, individuals in the younger age brackets (<85 years), city-dwelling residents, those living with others, and those from the most socioeconomically disadvantaged areas (deciles 1-3).

These results challenge traditional assumptions regarding the digital divide, specifically that low socioeconomic status and ICT ownership are barriers to telehealth adoption for older adults. Rather, our findings are consistent with arguments made by Dykgraaf et al [4], who suggest that for older people, digital literacy, trust, and familiarity are significant factors that can influence the adoption and use of telehealth. These results support our hypothesis and complement the findings by Mao et al [15] and Choi et al [12], affirming that health-related factors are critical determinants of telehealth adoption and use for older Australians. Individuals with a disability, chronic disease, multimorbidity and lower perceived quality of life and those experiencing missed or delayed care were more likely to exhibit telehealth use across all levels, highlighting the importance of telehealth as a supportive tool for managing complex health needs during the pandemic and out of pandemic conditions.

In addition, our findings have revealed that early or prior experience with telehealth is a significant predictor of its sustained use. Participants who engaged with telehealth before or early in the pandemic (as assessed in survey 1) were more likely to continue its use. This suggests that initial exposure and satisfaction with telehealth services are important predictors of adoption and long-term use for this population. The psychosocial factors of loneliness and psychological distress were linked to moderate levels of telehealth use, inferring that telehealth may have also played a role in mitigating mental health challenges by providing continued access to these health professionals.

Moreover, the study affirms the central concern that the expansion of telehealth services during the COVID-19 pandemic may have disproportionately benefited certain groups. For example, telehealth use was observed to be higher among those living in major cities, suggesting that older adults residing outside of city areas were less likely to adopt and use telehealth, potentially exacerbating health care disparities for those living in these underserviced geographic areas.

Although telehealth emerged as a supportive tool for many older people experiencing complex health conditions, it is important to acknowledge that a large proportion of the cohort did not adopt telehealth. A modest uptake of telehealth services was observed during the initial phase of the pandemic that was not sustained, declining over time even when ICT ownership was not a limiting factor. These findings are congruent with observations made by Haimi and Gesser-Edelsburg [6] and Lee et al [3], whereby increased telehealth service availability was not met by increased telehealth adoption or use. In particular, Lee et al [3] analyzed Australian Medicare data evaluating trends in telehealth and in-person health care visits (March 2020-2021). Similar to our findings, they observed an early surge in telehealth use during the introduction of initial lockdown restrictions with an associated decrease with in-person health care visits. Over time, in-person health care visits increased as telehealth visits decreased, suggesting a New South Wales population preference (personal and medical professional) for in-person health care [3].

Given our findings, further exploration of the interplay between sociodemographic and health-related factors as barriers and enablers to telehealth adoption use is warranted. Situating our results within the 4 domains of the Unified Theory of Acceptance and Use of Technology framework (ie, performance expectancy, effort expectancy, social influence, and facilitating conditions) may offer additional insights into an individual’s decision-making process related to telehealth adoption and integration into routine health care management [28].

### Performance Expectancy

Our findings emphasize the significant influence of health-related determinants on telehealth adoption and ongoing use. This aligns with the concept of performance expectancy, whereby a person’s decision to adopt a technology is influenced by the degree to which they believe the technology will help them [28]. Our findings indicate that an individual’s decision to adopt telehealth was significantly related to poorer health with those experiencing disability, chronic disease, or multimorbidity more likely to adopt and use telehealth across all levels. Moreover, experiencing missed or delayed care, experiencing psychological distress, and reporting a low perceived quality of life were also associated with moderate levels of telehealth use compared with those in the relevant reference groups. A systematic review by Wang et al [22] highlighted performance expectancy as a significant determinant of technology adoption among older adults. In this study, individuals reporting higher numbers of physical conditions and disability were found to be more willing to adopt long-distance caregiving technologies, including telehealth, to support their health and independence [22]. In addition, Mao et al [15] and Choi et al [12] similarly observed chronic conditions, multimorbidity, impairments with activities of daily living, and mental distress were determinants of telehealth use among their cohorts of older adults, further supporting the proposition that perceived health benefits drive technology adoption and use among older populations.

### Effort Expectancy

Our findings did not directly access effort expectancy, defined as perceived ease of use [28]; however, they do suggest an indirect relationship. Indicators, such as early or prior adoption and patterns of telehealth use, assist in explaining this construct. The logistic regression results revealed that early or prior exposure to telehealth (as assessed in survey 1) was a predictor of its sustained use in survey 5. Furthermore, in survey 1, approximately 50% of participants indicated that they probably would recommend telehealth to others, with an additional 21% indicating a definite willingness to endorse the use of telehealth services. These findings imply that initial, positive experiences with telehealth foster adoption. In addition, the high preference for telephone modes of telehealth (88.95% in survey 1) and low preference for video modes (3.16% in survey 1) suggests that familiarity with ICT mediums influences adoption. These observations collectively support the notion that perceived ease of use is a key factor influencing telehealth adoption among older adults.

Dykgraaf et al [4] emphasize the significance of ensuring that telehealth services are user-friendly in their design, supporting the need for telehealth platforms to use familiar technology and intuitive interfaces, particularly for enhancing usability by older people. Moreover, Choi et al [12] observed that ICT ownership and having the knowledge to use technology were significant enablers of telehealth use during the COVID-19 pandemic, suggesting that both familiarity with technology and the knowledge to use it are essential requisite skills required for adoption. This may be especially pertinent for those aged ≥85 years, who, in our analysis, were found to be less likely to exhibit high levels of telehealth use when compared with their younger peers. These results are again congruent with findings by Choi et al [12], whereby those of older age (>80 years) demonstrated decreased odds for telehealth use. Bridging this aspect of the digital divide is crucial, as a lack of ICT access and digital skills is a known barrier to telehealth adoption for older people [4,12]. However, according to Kruse et al [10] technology acceptance in older age groups may be more aligned with preferences for in-person health care, declaring that public policy may not help in this area and that health care providers may need to accept this preference.

### Social Influence

The role of social influence, defined as how individuals perceive the importance others place on using telehealth, also appears to play a role in an older person’s decision to adopt and use telehealth. In our analysis, individuals living with ≥1 other person were significantly more likely to adopt and have high use of telehealth compared with those who live alone (*P*<.001), suggesting an enabling effect. This could be through direct encouragement or through indirect influence of observing other’s positive experiences with telehealth. Wang et al [39] identified social support as a critical element in fostering telehealth adoption for older adults. A systematic review investigating factors influencing the acceptance of technology for aging in place additionally revealed the social environment as a determining factor [40]. In particular, the influence of children, health professionals, and caregivers was associated with technology adoption. The enabling effects of social influence on technology adoption are also suggested to increase as personal dependence on health care services increases, with older people being more open to influence by others, given their changing health circumstances [28].

In addition, from our analysis, approximately 50% of participants who used telehealth in survey 1 indicated that they probably would recommend telehealth to others, with an additional 21% indicating a definite willingness to endorse the use of telehealth. These findings suggest that a positive experience with telehealth may influence its social advocacy, with telehealth services being recommended to others. Similarly, poor experiences with telehealth may negatively affect the social advocacy of telehealth. For example, a mixed methods social media survey ascertained the experiences of telehealth use among a general population of Australians (>18 years, n=369) during the COVID-19 pandemic [9]. Findings from this study revealed that for many users, their telehealth experience was poor, as the service did not meet their health care needs and expectations. Contributing factors included a lack of visual cues, eye contact, and body language; an inability to be physically assessed; poor audio quality; poor connectivity; being seen by unknown health care providers; and feeling rushed when using telehealth services [9]. Experiences such as these will influence social advocacy for the use of telehealth services and are particularly relevant given that our findings indicate that approximately 24% of our sample that used telehealth probably will not and 3% definitely will not recommend telehealth services to someone else.

### Facilitating Conditions

Facilitating conditions, reflecting the degree to which a person believes that infrastructure exists to support their use of the technology, also influenced telehealth adoption [28]. Although the infrastructure required to support telehealth was in place and incentivized, it was less likely to be adopted or used by those living in inner regional, outer regional, rural, and very remote areas. It is well known that the IT infrastructure required to support telehealth varies in Australia especially for those living outside of city areas, thus limiting access [20]. Given that both moderate and high telehealth use were more likely to occur for participants living in a major city, it may be that underdeveloped digital technology infrastructure also led to disproportionate telehealth service access during this time [20].

### Implications of Findings

When considered alongside the principal findings, the expansion of telehealth services necessitates a nuanced understanding of the diverse factors that influence telehealth adoption and use behaviors, particularly for older Australians. This study revealed that high telehealth use was prevalent among women, those younger than 85 years, those residing within major cities, those who live with others, and those of socioeconomic disadvantage. The observed higher frequency of telehealth use among women compared with men suggests potential gender-related barriers to telehealth adoption. Gender differences in telehealth use have been noted in various studies. Choi et al [12] found that gender was not a significant predictor of telehealth use. In contrast, Mao et al [15] reported that women, especially those with chronic conditions and poorer mental health, were more likely to use telehealth compared with men. A more recent study by Haimi and Sergienko [41] also observed that women significantly increased their use of telehealth services during the COVID-19 pandemic across different types of telehealth services.

A recent rapid review by Turcotte et al [42] found age and gender to be moderating factors (barriers and enablers) for telehealth use, highlighting that older men, in particular, might face barriers related to mental health care that could affect their telehealth adoption. They noted that further research is required to understand gender differences in telehealth service use. To increase the frequency of telehealth adoption and use among older men, targeted strategies could include addressing specific concerns related to technology use, providing gender-sensitive training and support, and highlighting the benefits of telehealth for managing health conditions that are more prevalent among men. Encouraging health care providers to actively engage older male patients in discussions about telehealth options may also help increase adoption rates.

The provision of telehealth services for chronic disease and disability management shows promise as being associated with high telehealth use. Early or prior telehealth experiences that are perceived as positive and that use familiar technology require thoughtful consideration, potentially facilitating sustained use among this cohort. Telehealth technology is well positioned to overcome geographic barriers, particularly as specialist health care professionals in Australia, required to treat and manage chronic disease and disability, are concentrated within metropolitan areas [20]. However, significant investments are required to support information technology infrastructure for those living outside of city areas. As IT infrastructure improves, piloting specialist chronic disease telehealth models of care that link city specialists to those outside of these areas, using hybrid telehealth modes (combining in-person and telehealth), is one area for future research. To increase older adults’ willingness to adopt and use telehealth, subjective improvements, such as increasing awareness about the benefits of telehealth, providing user-friendly interfaces, and ensuring positive initial experiences with telehealth services, are essential. In addition, offering personalized assistance and training to build confidence in using telehealth technology may enhance the willingness to adopt telehealth into their nonpandemic health care routines.

### Limitations

The insights derived from this large-scale study on telehealth adoption and use during the COVID-19 pandemic by people aged ≥65 years are instrumental in understanding behavioral trends. However, the study’s cross-sectional nature limits our ability to infer causality despite identifying a temporal association between early telehealth use and sustained adoption. Self-reporting can introduce bias, potentially skewing the motivations behind telehealth use, especially given the variation in wording of questions across surveys. This is particularly relevant as, although we observed a higher reliance on telephone-based telehealth compared with video-based telehealth in the survey 1 time point, the question was not asked uniformly across surveys, and we could not provide trends of telephone and video telehealth use over time. Although the size of the cohort lends credibility to the generalizability of our findings, it does not fully capture individual longitudinal experiences or control for all potential confounding variables. In addition, the high level of technology literacy within our cohort might not reflect the broader older Australian population, who may encounter different challenges, as indicated by the underrepresentation of culturally and linguistically diverse participants. There is a clear need for longitudinal studies to trace the telehealth adoption journey over time and to consider a more diverse population to enhance the applicability of the findings.

### Conclusions

This study investigated the sociodemographic and health-related determinants influencing telehealth adoption and use among a cohort of older Australians during the COVID-19 pandemic. Our findings revealed that health-related characteristics, including those living with a disability, having a chronic disease, and multimorbidity, were significant predictors of telehealth adoption and ongoing use. Early or prior telehealth adoption was also found to be associated with its sustained use, independent of health-related factors, highlighting the importance of initial positive user experiences, familiarity with technology, and ease of use of the telehealth platforms required for this population. This research contributes to the broader understanding of telehealth adoption and use among older adults and highlights the necessity for targeted strategies to support its integration into routine health care delivery for older adults. As the world continues to navigate the pandemic and witnesses the increasing prevalence of noncommunicable chronic disease, our findings provide a foundation for policymakers and health care providers to optimize telehealth services, thereby promoting equitable health care access and supporting the well-being of older populations.

## Abbreviations

CALD: cultural and linguistic diversity
ICT: information and communication technology

## Data Availability

The data sets analyzed for this study are not publicly available as they are privately owned and accessed through The Sax Institute. All data analyzed during this study are included in this published article.
